# VHPKQHR Peptide Modified Ultrasmall Paramagnetic Iron Oxide Nanoparticles Targeting Rheumatoid Arthritis for T_1_-Weighted Magnetic Resonance Imaging

**DOI:** 10.3389/fbioe.2022.821256

**Published:** 2022-02-28

**Authors:** Chunyu Zhang, Wentao Huang, Chen Huang, Chengqian Zhou, Yukuan Tang, Wei Wei, Yongsheng Li, Yukuan Tang, Yu Luo, Quan Zhou, Wenli Chen

**Affiliations:** ^1^ MOE Key Laboratory of Laser Life Science, Institute of Laser Life Science, College of Biophotonics, South China Normal University, Guangzhou, China; ^2^ Guangdong Provincial Key Laboratory of Laser Life Science, College of Biophotonics, South China Normal University, Guangzhou, China; ^3^ Guangzhou Key Laboratory of Spectral Analysis and Functional Probes, College of Biophotonics, South China Normal University, Guangzhou, China; ^4^ Department of Minimally Invasive Interventional Radiology, Guangzhou Panyu Central Hospital, Guangzhou, China; ^5^ Neuroscience Laboratory, Hugo Moser Research Institute at Kennedy Krieger, Baltimore, MD, United States; ^6^ Institution of GuangDong Cord Blood Bank, Guangzhou, China; ^7^ Shanghai Engineering Technology Research Center for Pharmaceutical Intelligent Equipment, Shanghai Frontiers Science Research Center for Druggability of Cardiovascular Noncoding RNA, Institute for Frontier Medical Technology, College of Chemistry and Chemical Engineering, Shanghai University of Engineering Science, Shanghai, China; ^8^ Department of Radiology, The Third Affiliated Hospital of Southern Medical University, Guangzhou, China

**Keywords:** rheumatoid arthritis, magnetic resonance imaging, USPIO, VCAM-1, UVHP

## Abstract

Magnetic resonance imaging (MRI) could be the ideal diagnostic modality for early rheumatoid arthritis (RA). Vascular cell adhesion molecule-1 (VCAM-1) is highly expressed in synovial locations in patients with RA, which could be a potential target protein for RA diagnosis. The peptide VHPKQHR (VHP) has a high affinity to VCAM-1. To make the contrast agent to target RA at an early stage, we used VHP and ultrasmall paramagnetic iron oxide (USPIO) to synthesize UVHP (U stands for USPIO) through a chemical reaction with 1-(3-dimethylaminopropyl)-3-ethylcarbodiimide hydrochloride and N-hydroxysuccinimide. The size of UVHP was 6.7 nm; the potential was −27.7 mV, and the *r*_2_/*r*_1_ value was 1.73. Cytotoxicity assay exhibited that the cell survival rate was higher than 80% at even high concentrations of UVHP (Fe concentration 200 µg/mL), which showed the UVHP has low toxicity. Compared with no TNF-α stimulation, VCAM-1 expression was increased nearly 3-fold when mouse aortic endothelial cells (MAECs) were stimulated with 50 ng/mL TNF-α; cellular Fe uptake was increased very significantly with increasing UVHP concentration under TNF-α treatment; cellular Fe content was 17 times higher under UVHP with Fe concentration 200 µg/mL treating MAECs. These results indicate that UVHP can target overexpression of VCAM-1 at the cellular level. RA mice models were constructed with adjuvant-induced arthritis. *In vivo* MRI and biodistribution results show that the signal intensity of knee joints was increased significantly and Fe accumulation in RA model mice compared with normal wild-type mice after injecting UVHP 24 h. These results suggest that we have synthesized a simple, low-cost, and less toxic contrast agent UVHP, which targeted VCAM-1 for early-stage RA diagnosis and generates high contrast in T_1_-weighted MRI.

## 1 Introduction

Rheumatoid arthritis (RA), a chronic systemic immune disease (Firestein, 2003), has a global prevalence of 0.5% to 1% (Abdel-Nasser et al., 1997; Kvien, 2004). Early diagnosis and correct treatment can effectively alleviate the incidence of RA patients (Luo et al., 2018). Conventional radiography, computed tomography, ultrasonography, and magnetic resonance imaging (MRI) are common imaging methods for RA (Østergaard et al., 2008). Because of the high resolution of the tissue, MRI may be one of the most ideal methods used clinically for the early diagnosis of RA (Østergaard et al., 2008; Hou et al., 2020).

MRI contrast agents are divided into longitudinal relaxation contrast agents (T_1_-weighted contrast agents) and transverse relaxation contrast agents (T_2_-weighted contrast agents) according to their principle of action (Zhao et al., 2021). T_1_-weighted contrast agent shortens the longitudinal relaxation time of the tissue and enhances the signal; T_2_-weighted contrast agent shortens the transverse relaxation time of the tissue and weakens the signal. As the T_2_ signal is dark, and T_1_ is a bright signal, and the high magnetic moment of the T_2_ contrast agent may cause magnetic-susceptibility artifacts (Na et al., 2009; Zhou et al., 2020), and human vision prefers bright signals; T_1_ contrast agent is more prevalently used than T_2_ in a clinical setting.

Commonly used clinical T_1_ contrast agents include Gd-DTPA (gadopentetate meglumine) (Carr et al., 1984), Gd-DOTA (Dotarem) (Meyer et al., 1988), and other Gd-type contrast agents, and Mn-DPDP (mangafodipir) (Jynge et al., 2020), MnCl_2_, and other Mn contrast agents. However, these contrast agents are not selective, and Gd-based contrast agents may be nephrotoxic (Prince et al., 2008). Because contrast agents can improve the sensitivity of MRI (Caravan, 2006), it is necessary to find a contrast agent for early targeted diagnosis of RA.

Superparamagnetic iron oxide nanoparticles (SPIO) and ultrasmall SPIO (USPIO) are T_2_ contrast agents that have been used in the clinic (Zhao et al., 2021). USPIO exhibits a decrease in magnetization due to the spin tilt effect as the particle size decreases, which effectively shortens the T_1_ relaxation time of water protons. So, the USPIO can be used to improve T_1_-weighted MRI signals (Chen et al., 2020). USPIO with a particle size of less than 5 nm has received more and more attention from researchers as MRI T_1_ contrast agents (Zhou et al., 2020).

Simon et al. compared the imaging performance of 3.0 nm USPIO coated with carboxyl glucose and GD-DTPA for arthritis. The study shows that USPIO has a higher blood half-life and a more significant T_1_ contrast (Simon et al., 2006). Li et al. designed a T_1_/T_2_-weighted contrast agent light-addressable ultrasmall Fe_3_O_4_ nanoparticles, which targeted macrophages in arthritis by folic acid. After 405-nm laser irradiation, the nanoparticle forms nanoclusters leading to T_1_-weighted MRI change into T_2_-weighted MRI (Li et al., 2019).

The specific mechanism of RA is currently unknown, but many clinical phenotypes of RA have been reported, including inflammatory factors (such as tumor necrosis factor-α [TNF-α] and interleukin-8) in the joint environment of RA patients; these inflammatory factors lead to the vascular cell adhesion molecule-1 (VCAM-1) overexpression in the synovium around the joint. Overexpression VCAM-1 was observed in fibroblast-like synoviocytes and endothelial cells (Elices et al., 1992; Dolati et al., 2016). VHPKQHR (VHP) is a peptide targeted to VCAM-1 identified by phage display technology and has been applied to target atherosclerotic plaque (Nahrendorf et al., 2006; Xu et al., 2019), but the use of VHP combined with USPIO for RA targeted imaging has not been reported yet.

This study synthesized a T_1_-weighted MRI contrast agent UVHP (USPIO combined with VHP) that can target RA. Because of its low cost, simple method, and low toxicity, UVHP has the potential for clinical application.

## 2 Materials and Methods

### 2.1 Synthesis and Characterization of USPIO and UVHP

#### 2.1.1 Synthesis of USPIO

Synthesis of USPIO by the method provided in the published research article (Luo et al., 2015) was performed as follows: sodium citrate (final concentration 45.60 mM) was added to 80 mL of homogeneous FeCl_3_ solution (dissolved in the final concentration of diethylene glycol 81.77 mM) in a water bath at 80°C until a clarified solution was formed. Subsequently, sodium acetate (final concentration 299.06 mM) was added to the above mixture and dissolved, and then the mixture was transferred to a 100-mL Teflon-lined stainless-steel autoclave reactor and sealed. The autoclave was placed in an oven at 200°C for 4 h. After cooling to room temperature, the black precipitate was collected by centrifugation (13,200*g*, 15 min). Precipitation was washed three times with ethanol to remove excess reactants and by-products then were dissolved in 10 mL ddH_2_O and lyophilized to obtain 460 mg powder for the next step.

#### 2.1.2 Synthesis of UVHP

USPIO (100 mg) was dissolved in 18 mL of ddH_2_O at room temperature. 1-(3-Dimethylaminopropyl)-3-ethylcarbodiimide (molecular weight [MW] = 192; Shanghai Yuanye Bio-Technology Co., Ltd.) in 1 mL ddH_2_O (final concentration 0.047 M) and N-hydroxysuccinimide (MW = 115.8; Shanghai Yuanye Bio-Technology Co., Ltd.) in 1 mL H_2_O (final concentration 0.03 M) were added to the reaction solution and activated by vigorous stirring under ice bath conditions for 6 h. The peptide (VHPKQHR, Sangon Biotech [Shanghai] Co., Ltd.) 20 mg (MW = 1,081) was added and reacted for 6 h under an ice bath. The reaction was carried out with a dialysis membrane (MW cutoff = 1,500) in phosphate-buffered saline (PBS, three times, 2 L) and ddH_2_O (six times, 2 L) to dialyze the mixture for 3 days under an ice bath to remove unreacted excess reactants. After freeze-drying, 83.4 mg UVHP was obtained (U stands for USPIO, VHP stands for VHPKQHR).

#### 2.1.3 Characterization of Nanoparticles

Fourier transform infrared (FTIR) spectra were measured on a Bio-Rad FTS 6000 spectrometer (Bio-Rad Company, Hercules, CA, USA) at 25°C (Li et al., 2020). Thermogravimetry (TGA) curves were carried out using a NETZSCH thermal analyzer (DSC 204F1) at a heating rate of 10°C/min in a nitrogen atmosphere (Yu et al., 2015). Zeta potential and dynamic light scattering (DLS) measurements were conducted using a Malvern Zetasizer Nano ZS (Malvern Instruments) (Li et al., 2020). The morphology and size of USPIO and UVHP were characterized by a field emission 2100F transmission electron microscope (TEM; JEOL, Japan) under 200-kV acceleration voltage (Yang et al., 2021). One-tesla MRI scanner (NM42-040H-I, Niumag, China) was used to measure the *r*_1_ and *r*_2_ values of USPIO and UVHP. The Fe contents of USPIO and UVHP were detected by ICP-OES (inductively coupled plasma optical emission spectrometer; Thermo E. IRIS Duo) (Tao et al., 2019). For example, we weighed 2,000 µg UVHP, and after measuring with ICP-OES, the Fe content was 262 µg. According to this result, we can weigh the corresponding UVHP amount according to the Fe content required by our experiments (Supplemental data). T_1_ relaxometry and T_2_ relaxometry were performed by a 0.5-T NMR analyzer (MINIPQ001; Shanghai Niumag Co., China).

### 2.2 Material, Cell Cultivation, and Sample Processing

#### 2.2.1 Cell Material and Culture

Mouse aortic endothelial cells (MAECs) were purchased from CHI Scientific, Inc. Raw 264.7 cell line was obtained from ATCC (American Type Culture Collection). Human fibroblast-like synoviocyte RA (HFLS-RA) cell line was purchased from Guangzhou Jennio-bio Co., Ltd. (Guangzhou, China). MAECs, HFLS-RA cells, and raw 264.7 cells were grown in Dulbecco modified eagle medium (DMEM) medium containing 10% fetal bovine serum and 1% penicillin–streptomycin at 37°C in a humidified atmosphere with 5% CO_2_.

#### 2.2.2 Animal Model and Culture

Two-month-old female C57BL/6 mice were purchased from Animal Experimental Center, Southern Medical University. The mice had free access to food and water under a 12-h light/12-h dark cycle. All animal experiments were approved by the Institutional Animal Ethics Committee of South China Normal University and were carried out following the National Institutes of Health Guidelines for the Care and Use of Laboratory Animals of South China Normal University.

C57BL/6 mice were used to construct RA model mice. Complete Freund’s adjuvant was mixed with 2% methylated bovine serum albumin (mBSA) in equal volume and injected into the axillae of 2-month-old female C57BL/6 mice at 0.1 mL per side. Three weeks later, 2% mBSA solution (dissolved in sterile water) was injected into the joint cavity of mice at 10 µL per side (Jones et al., 2018). Two months later, RA model (antigen-induced arthritis) mice were molded. In animal experiments, C57BL/6 mice of the same sex and age were used as wild-type (WT) mice. RA model mice identification: RA model mice (n = 3) were euthanized, and the knee joints were fixed with formalin and were sent to Servicebio (Wuhan, China) for hematoxylin and eosin (H&E) staining, with C57BL/6 mice (n = 3) as controls. Safranin O/fast green (safranin/O) staining was carried out with the modified Safranin O-Fast Green FCF Cartilage Stain Kit (Solarbio, Beijing, China). The histological scoring of the sections was performed by three unwitting researchers using the Mankin scoring system. According to the classification of Mankin and his colleagues, the score is divided into four stages: near normal (0–2), early (2–6), middle (6–10), and late (10–14) (Iannitti et al., 2013; Nishitani et al., 2014).

#### 2.2.3 Hemolytic Assay

Fresh blood from 8-week-old C57BL/6 mice is added to a tube containing sodium heparin. The erythrocyte was collected by centrifugation at 650*g* for 10 min. Then, erythrocyte was diluted to 0.25% with PBS (pH 7.4) and incubated with different concentrations of UVHP (Fe concentration 12.5, 25, 50, 100, 200 µg/mL) at 37°C for 12 h. Erythrocyte diluted with deionized water was used as a negative control, and erythrocyte diluted with PBS was used as a positive control. After 12 h of treatment, each group was centrifuged at 10,000*g* for 5 min, and 200 µL of supernatant was placed in a 96-well plate. The absorbance at 542 nm was measured using a microplate reader (INFINITE M200, Tecan, Switzerland). Each group was repeated three times.

#### 2.2.4 Cytotoxicity Assay

The cytotoxicity of UVHP on RAW264.7 and HFLS-RA cells was investigated by the Cell Counting Kit-8 (CCK-8; Dojindo Laboratories, Kumamoto, Japan) method. The cells were inoculated at a cell density of 1 × 10^4^ per well in 96-well plates and were cultured for 12 h or 24 h with a fresh medium containing different concentrations of UVHP (Fe concentration 0, 5, 10, 25, 50, 100, 150, 200 µg/mL) instead of nutrient-depleted medium, followed by 2 h with fresh serum-free medium containing 10% CCK-8. The absorbance at 450 nm in each well was measured using a microplate reader (INFINITE M200, Tecan, Switzerland); cell viability was calculated according to the instructions. All experiments were performed three times in parallel.

#### 2.2.5 Toxicity Evaluation

The UVHP (Fe content 150 µg, dissolved in 100 µL PBS) was injected into RA mice (n = 3) through the tail vein. After 14 days, organs (heart, liver, spleen, lungs, kidneys) were collected from the mice. Sections and H&E staining were obtained from Servicebio (Wuhan, China) after 24 h of fixation with formalin. The RA mice (n = 3) injected with saline served as controls.

#### 2.2.6 Western Blot

We treated MAECs with TNF-α (Abbkine Scientific Co., Ltd.) at concentrations of 10 and 50 ng/mL for 24 h (Fan et al., 2019); the cell culture medium was removed and washed three times with PBS. The cells were lysed by adding cell lysate on ice for 30 min and then quickly scraped with a clean cell scraper. Cell debris and lysate were centrifuged at 13,200*g* for 20 min, and then the supernatant was collected. Protein samples were denatured and separated on sodium dodecyl sulfate–polyacrylamide gels and then transferred to polyvinylidene difluoride membranes (cat. no.: IPVH00010 PORE SIZE: 0.45 µm; Merck Millipore Ltd.). The membranes were blocked with TBST solution (150 mM NaCl, 10 mM Tris–HCl, pH 7.4, 0.1% Tween 20) containing 5% skim milk for 2 h, followed by incubation with specific primary antibody (anti–VCAM-1 polyclonal antibody, Servicebio; anti-GAPDH [GAPDH was used as loading control], Absin, Shanghai, China) and secondary antibody (goat anti-rabbit immunoglobulin G [IgG, corresponding to VCAM-1]; MultiSciences, Hangzhou, China; goat anti-mouse IgG [corresponding to GAPDH], Dingguo, Beijing, China). Signals were detected using an image analyzer (Tanon-5200, Shanghai, China). The experiment was repeated three times, and results were analyzed using ImageJ software.

#### 2.2.7 The *In Vivo* Biodistribution of UVHP

The mice after scanning with MRI were euthanized, and the organs heart, liver, spleen, lungs, kidneys, and knee joints were extracted and weighed, respectively. They were cut into small pieces of 1 to 2 mm and digested with aqua regia solution for 24 h, and then ICP-OES was used to detect the Fe content of different organs (Hou et al., 2020).

#### 2.2.8 Cell Targeting MRI Experiment of UVHP

Cells (MAECS or HFLS-RA cells) were seeded at 1 × 10^5^ cells per well in six-well plates with DMEM, and TNF-α (50 ng/mL for MAECs and 10 ng/mL for HFLS-RA [Croft et al., 1999]) or the same volume of PBS (as a control) was added, respectively. After 24 h of incubation, DMEM containing USPIO, UVHP (both Fe 50 µg/mL), or DMEM (as a control) replaced the original cell culture medium, treatment for 24 h. Raw 264.7 cells were also seeded at 1 × 10^5^ cells per well in six-well plates with DMEM. After 24 h, DMEM containing USPIO, UVHP (both Fe 50 µg/mL), or DMEM (as a control) was used to replace the original cell culture medium for 24 h of treatment. The cells were washed three times with PBS to remove USPIO/UVHP that were not phagocytosed by the cells and then collected by trypsin and dispersed in 0.5 mL agarose (1%). MRI scan was obtained with a Philips 3.0 T Achieva MRI system (Healthcare, Best, the Netherlands), with the following scan parameters: FOV (field of view) = 60 × 60 mm, matrix = 256 × 256, slice thickness = 2 mm, TR (time of repetition) = 1,200 ms, and TE (time of echo) = 17 ms.

#### 2.2.9 Cell Targeting Quantitative Determination Experiment of UVHP

Cells (MAECS or HFLS-RA cells) were seeded in six-well plates at a density of 1 × 10^5^ and treated with TNF-α (50 ng/mL) for 24 h. The culture medium was replaced by a fresh culture medium containing UVHP (Fe concentration 25, 50, 100, and 200 µg/mL) and incubated for 24 h. The cells were washed three times with 1 mL PBS to remove the UVHP that were not taken up by the cells and then trypsinized and collected by centrifugation. The cells were resuspended with 1 mL PBS; the number of cells per well was counted, and then the cells were digested with aqua regia (Luo et al., 2015). ICP-OES was used to detect the Fe content of MAECs. For comparison, the same experiments were performed with cells (MAECS or HFLS-RA cells) treated with PBS (instead of TNF-α) for 24 h, and the subsequent UVHP treatment of cells and other operations are the same.

#### 2.2.10 *In Vivo* MRI of UVHP Targeting RA

The knee joints of RA mice (n = 3) and C57BL/6 mice (n = 3) were scanned with a 7-T MRI (Bruker Biospec 7 T), with the following scan parameters: TR = 900, TE = 9 ms, FOV = 80 × 80 mm, matrix = 256 × 256, ST (slice thickness) = 1 mm.

Two groups of RA mice (n = 3) were selected and injected with USPIO, UVHP (both Fe content 150 µg, dissolved in 100 µL PBS) in the tail vein, respectively. Two groups of C57BL/6 mice (n = 3) were selected and treated with the same conditions as controls. The mice were scanned after injecting UVHP for 24 h. T_1_-weighted imaging was performed with a 7-T MRI scanner (Bruker Biospec 7 T). Scan parameters were as follows: TR = 1,000, TE = 8.5 ms, FOV = 80 × 80 mm, matrix = 256 × 160, ST = 1 mm. For image analysis, all images were analyzed via MRI viewing station (Paravision 5.1; Bruker, Karlsruhe, Germany).

#### 2.2.11 Statistical Analysis

The significant difference of the experimental data was performed by one-way analysis-of-variance statistical analysis. When *p* < 0.05, the values were considered to be statistically significantly different. *p* < 0.05 is represented by (*), *p* < 0.01 is represented by (**), and *p* < 0.001 is represented by (***).

## 3 Results

### 3.1 Synthesis and Characterization of USPIO and UVHP

**FIGURE 1:**
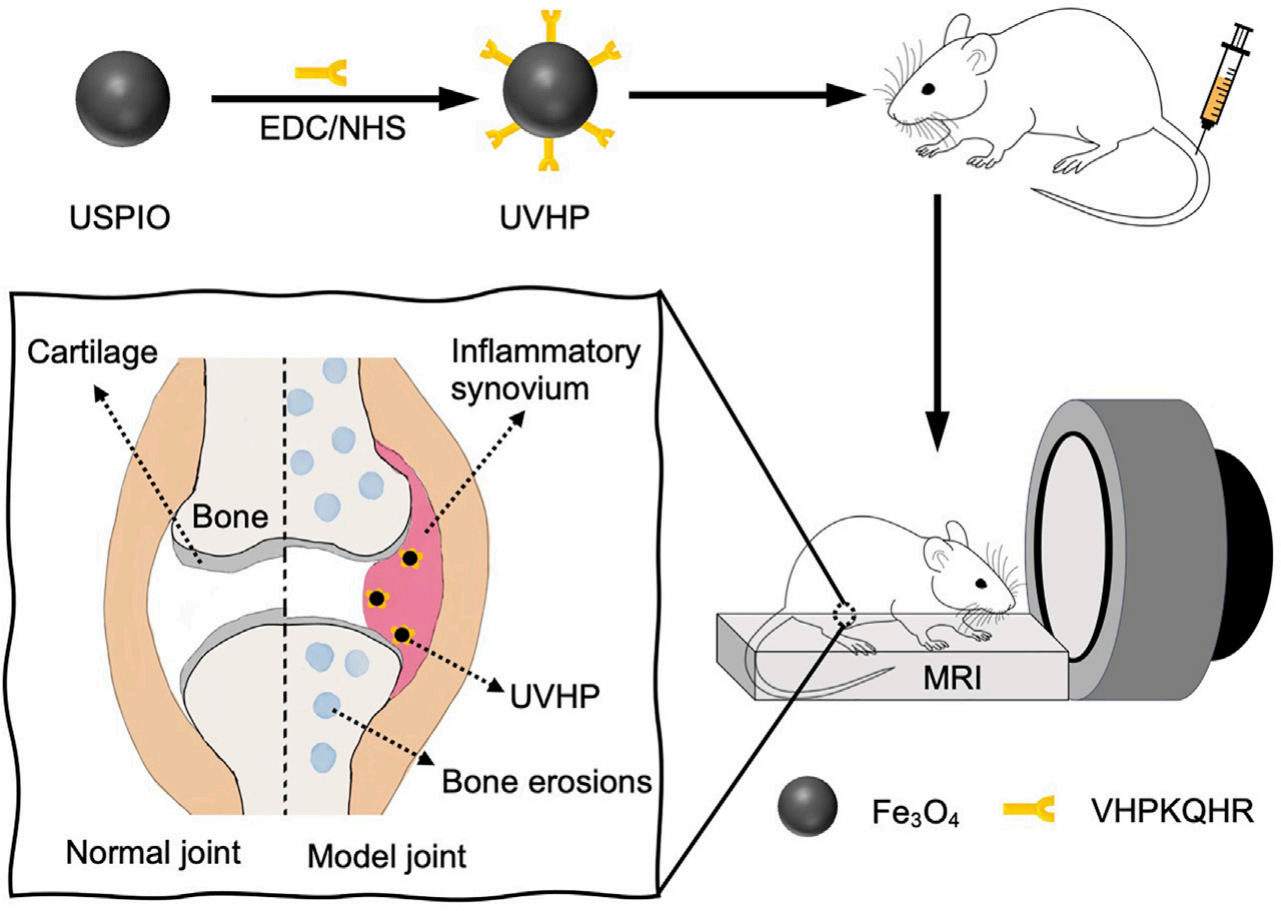
Schematic illustration of the preparation and targeting rheumatoid arthritis of UVHP.

**FIGURE 2:**
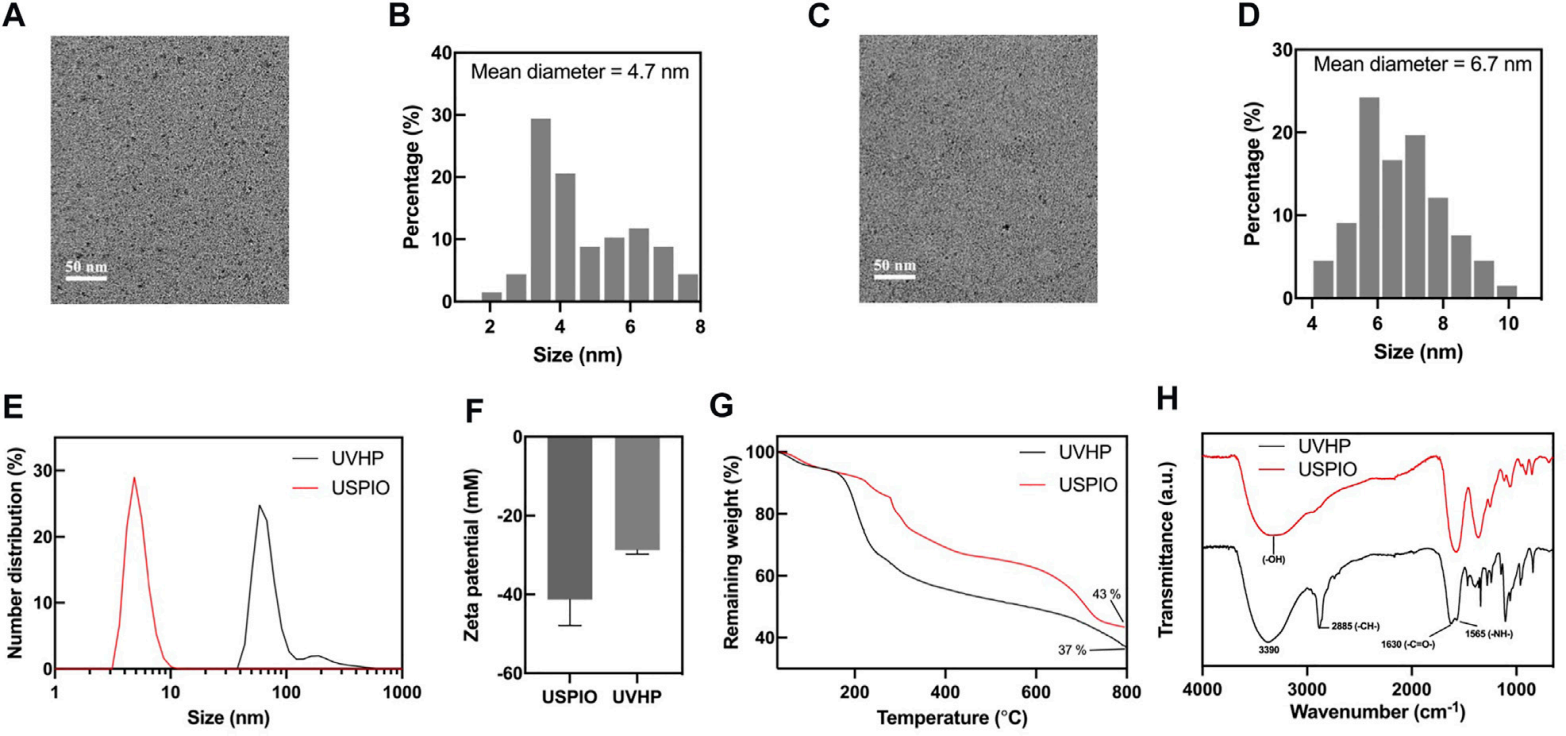
Characterizations of USPIO and UVHP. **(A)** TEM image of USPIO; **(B)** size distribution histogram of USPIO; **(C)** TEM image of UVHP; **(D)** size distribution histogram of UVHP; **(E)** hydrodynamic sizes of USPIO and UVHP; **(F)** zeta potentials of USPIO, UVHP; **(G)** TGA curves of USPIO, UVHP; **(H)** FTIR spectra of USPIO, UVHP.

We synthesized surface carboxylated USPIO according to the articles by Chen et al. (2020) and Luo et al. (2015) (for more details, see *Materials and Methods*). After that, we linked peptides VHP and USPIO together with an amide bond to form UVHP (Figure 1) (for more details, see *Materials and Methods*). TEM was used to characterize the size and morphology of USPIO and UVHP (Figures 2A,C). The size distribution histogram showed that the mean diameter of the USPIO was 4.7 ± 2.76 nm (Figure 2B); the zeta potential was −40 mV. Compared with USPIO, the particle size of the UVHP increased to 6.69 ± 2.78 nm (Figure 2D), and the zeta potential changed to −27.7 mV (Figure 2F), which should be attributed to the VHP connecting to the USPIO surface. DLS analysis showed that the hydrodynamic sizes of USPIO and UVHP were approximately 4 and 60 nm, respectively (Figure 2E). The increase in the hydrodynamic size of UVHP may be due to the decrease in the surface potential of the particles after the VHP was connected, which reduced the repulsion between the particles (Liu et al., 2013).

The combination of peptide VHP with USPIO was further confirmed by Fourier FTIR. As shown in Figure 2H, the absorption peaks in FTIR spectra at 3,390 cm^−1^, 2,885 cm^−1^, 1,630 cm^−1^, and 1,565 cm^−1^ represent the O–H, C–H, and N–H stretching of UVHP, which could be attributed to the successful connection VHP with USPIO. The VHP conjugated to the surface of USPIO was quantitatively characterized by TGA (Figure 2G). The USPIO had a weight loss of 57% (Figure 2G), which should be attributed to the citrate on the particle surface. The weight loss of UVHP was 63% after modification with the peptide VHP. The above results indicate that we successfully synthesized UVHP.

### 3.2 T_1_ and T_2_ Relaxometry and MRI of USPIO and UVHP

**FIGURE 3:**
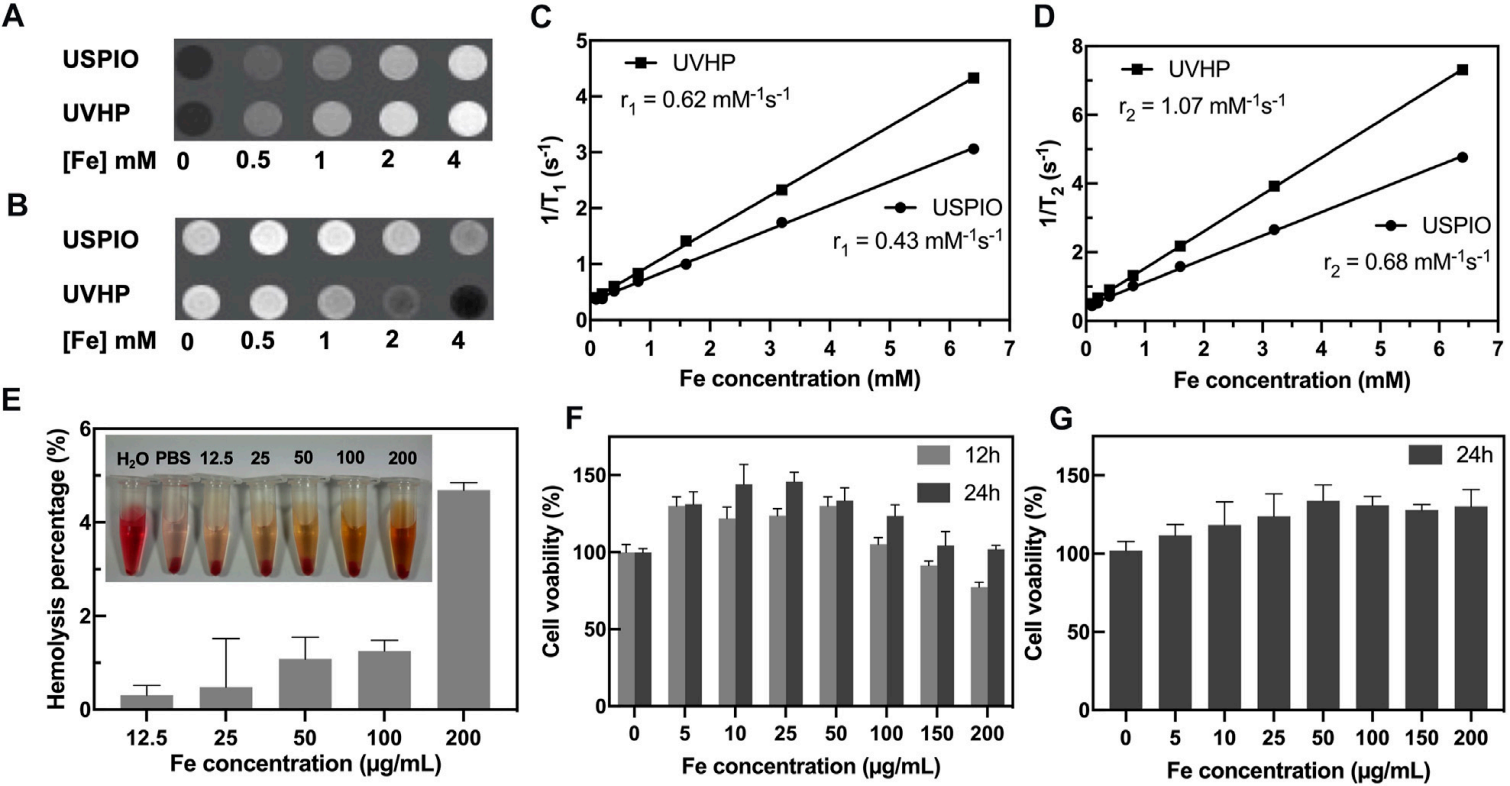
MRI scans and cytotoxicity assay of UVHP. T_1_-weighted **(A)** and T_2_-weighted **(B)** images of UVHP aqueous solution at different Fe concentrations (0, 0.5, 1, 2, and 4 mM) taken by a 3.0-T clinical MRI system under the following parameters: FOV = 100 mm; matrix = 168 × 168; section thickness = 2 mm; TR = 2,000 ms; TE = 60 ms. USPIO was used as control. **(C,D)** Plot of 1/T_1_ and 1/T_2_ over Fe concentration of UVHP, the slope indicates the specific relaxivity (*r*_1_ and *r*_2_). USPIO was used as control. **(E)** The hemolysis assay of UVHP (Fe concentration 12.5, 25, 50, 100, 200 µg/mL). PBS and water were used as negative and positive controls. **(F)** The viability of RAW 264.7 cells after coculture with UVHP at different Fe concentrations for 12 or 24 h was measured by the CCK8 assay. RAW 264.7 cells treated with PBS were used as control, respectively. **(G)** The viability of HFLS-RA cells after coculture with UVHP at different Fe concentrations for 24 h was measured by the CCK8 assay. HFLS-RA cells treated with PBS were used as control, respectively.

To test the potential of UVHP in MRI application, we monitor the MRI signal intensity of T_1_-weighted MRI and T_2_-weighted MRI of USPIO and UVHP using a 3.0-T clinical MRI system. The MRI results (Figure 3A) showed that as the Fe concentration increased, the T_1_-weighted MRI scan became brighter, and UVHP would be slightly brighter than USPIO under the same Fe concentration. The T_2_-weighted MRI (Figure 3B) showed that the image became darker as the Fe concentration increased; UVHP would be slightly darker compared with USPIO under the same Fe concentration (Figure 3B). Furthermore, when picking relaxation rate (1/T_1_ or 1/T_2_) as the ordinate and Fe concentration as the abscissa, Fe concentration is linearly proportional to the value of 1/T_1_ or 1/T_2_ (Luo et al., 2015), and their slope was the longitudinal relaxation rate *r*_1_ and transverse relaxation rate *r*_2_ values of USPIO and UVHP, respectively (Figures 3C, D). The *r*_1_ and *r*_2_ values of USPIO were 0.43 and 0.68 mM^−1^s^−1^, respectively; the *r*_2_/*r*_1_ ratio was 1.58. As for UVHP, the *r*_1_ and *r*_2_ values were 0.62 and 1.07 mM^−1^s^−1^, respectively; the *r*_2_/*r*_1_ ratio was 1.73. T_1_ contrast agent should have a high *r*_1_ relaxation rate and low *r*_2_/*r*_1_ value, and theoretically, *r*_2_/*r*_1_ value in the range of 1 to 2 should be suitable for T1-weighted MRI (Tromsdorf et al., 2009). Combining T_1_-weighted MRI and the value of *r*_2_/*r*_1_, USPIO and UVHP could be used as a potential T_1_-weighted imaging contrast agent in the future.

### 3.3 Hemolysis and Cytotoxicity Assay of UVHP

Hemolytic assay was used to investigate the hemocompatibility of the UVHP. As shown in Figure 3E, there was no significant hemolysis phenomenon observed for blood cells treated with UVHP with Fe concentrations of 12.5, 25, 50, 100, and 200 µg/mL. Further quantitative analysis showed that the hemolytic percentages of UVHP (Fe concentrations 0–200 µg/mL) were all lower than 5% (a threshold value) (Hu et al., 2015).

The cytotoxicity of UVHP was evaluated by CCK-8 before *in vivo* biomedical applications (Figure 3F). After incubation with the UVHP at Fe concentrations of 0, 5, 10, 25, 50, 100, 150, and 200 µg/mL for 12 h, the cell viability of RAW264.7 was approximately 80% at 200 µg/mL Fe concentration. Most investigators chose 80% as the threshold for cytotoxicity (Chen et al., 2020). Next, we extended the treatment time to 24 h; as Figure 3F shows, the cell survival rate was still greater than 80% when Fe concentration was 200 µg/mL. In order to further investigate the cytotoxicity of UVHP in RA, we incubated HFLS-RA cells with UVHP for 24 h, and the result of cell viability (Figure 3G) was similar to that of RAW264.7 cells. These results indicate that UVHP had low toxicity and hemocompatibility in Fe concentration of 0 to 200 µg/mL.

### 3.4 UVHP Targeting Cells (MAECs or HFLS-RA Cells) of TNF-α Stimulation

**FIGURE 4:**
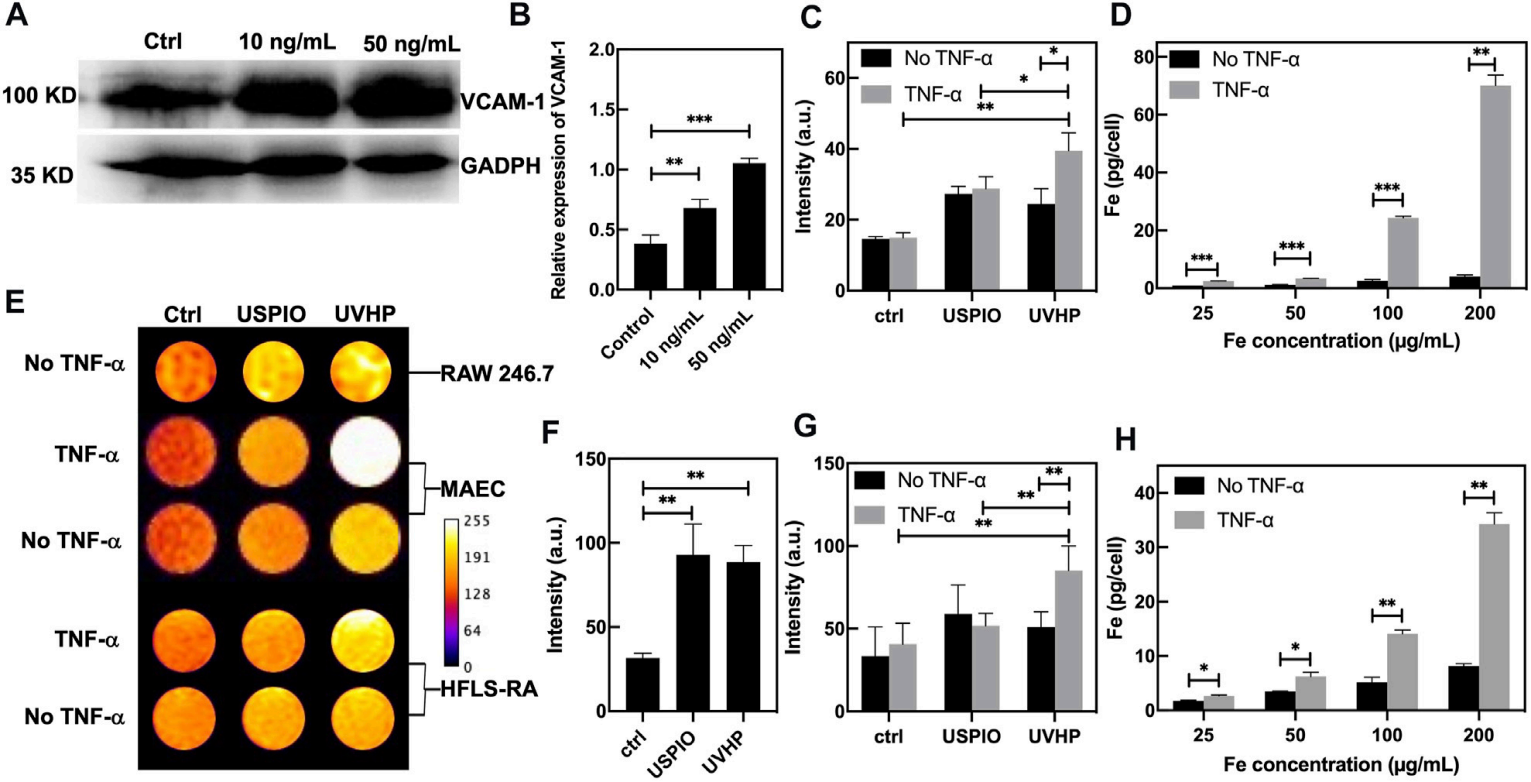
TNF-α–stimulated MAEC high expression of VCAM-1 and cell targeting assay. **(A)** Western blot of MAECs treated with TNF-α (10 ng/mL, 50 ng/mL). MAECs treated with PBS were used as control. **(B)** Quantitative Western blot analysis of VCAM-1. **(C)** T_1_-weighted MRI signal values of non–TNF-α–stimulated MAECs and TNF-α–stimulated MAECs treated with the USPIO and UVHP (both Fe concentration 50 µg/mL). **(D)** The TNF-α–stimulated MAEC uptake of the UVHP with different concentrations (Fe concentration 25, 50, 100, and 200 µg/mL) for 24 h. No TNF-α–stimulated MAECs were used as control. **(E)** T_1_-weighted MR pseudocolor images of cells. Raw 264.7 cells treated with USPIO or UVHP (both Fe concentration 50 µg/mL) for 24 h. MAECs treated with USPIO or UVHP (both Fe concentration 50 µg/mL) for 24 h in the presence or absence of TNF-α (50 ng/mL). HFLS-RA cells treated with USPIO or UVHP (both Fe concentration 50 µg/mL) for 24 h in the presence or absence of TNF-α (10 ng/mL). All cells treated with PBS as controls. **(F)** T_1_-weighted MRI signal values of non–TNF-α–stimulated raw 264.7 cells treated with the USPIO and UVHP (both Fe concentration 50 µg/mL). **(G)** T_1_-weighted MRI signal values of non–TNF-α–stimulated HFLS-RA cells and TNF-α–stimulated HFLS-RA cells treated with the USPIO and UVHP (both Fe concentration 50 µg/mL). **(H)** The TNF-α–stimulated HFLS-RA cell uptake of the UVHP with different concentrations (Fe concentration 25, 50, 100, and 200 µg/mL) for 24 h. Non–TNF-α–stimulated HFLS-RA cells were used as control.

It has been previously reported that TNF-α is highly expressed in RA lesions (Choy and Panayi, 2001; da Costa Moura, 2013), and VCAM-1 overexpression was observed in MAECs under stimulation with TNF-α (Fan et al., 2019). So, we chose MAECs with TNF-α treatment to mimic the microenvironment at the site of arthritis. As shown in Figure 4A, compared with the MAECs treated with PBS (as control), the expression of VCAM-1 was increased after MAECs treated with TNF-α for 24 h. The Western blot analysis demonstrated that VCAM-1 expression in MAECs was increased two and three times after 10 and 50 ng/mL TNF-α stimulation, respectively, comparing with the control (Figure 4B). These results confirm that TNF-α could increase the VCAM-1 expression in MAECs.

First, we used MRI to explore the effect of UVHP on RAW264.7 cells. After 24 h of UVHP or USPIO treatment, RAW264.7 cells were examined under T_1_-weighted MRI, and the signal intensity was counted (Figure 4E, first row). The results (Figure 4F) showed that there was no significant difference in the signal intensity between cells treated with UVHP and that, after USPIO treatment, both were higher than those of PBS-treated cells.

In order to explore the ability of UVHP to target VCAM-1, we used UVHP to treat MAECs and HFLS-RA cells, respectively. As shown in Figure 4E, we set up a PBS experimental group and a TNF-α experimental group for each cell type, which treated them with PBS or TNF-α for 24 h, respectively. Each group contained three different cell treatment subgroups: PBS, UVHP, or USPIO (both Fe concentration 50 ng/mL); for 24 h, the T_1_-weighted MRI signal intensity was counted (Figure 4E, second to fifth row). The signal of the statistical results is shown in Figure 4C and Figure 4G. In the PBS group (no TNF-α treatment), there was no significant difference in the signals after UVHP and USPIO treated MAECs and HFLS-RA cells, and both were higher than those treated with PBS. These results show that both USPIO and UVHP enhanced the T1-weighted signal strength, which are similar to the results of the RAW264.7 cells experiment above. In the TNF-α treatment group, the T_1_-weighted MRI signal after UVHP treatment was significantly higher than that of the USPIO group, and both were significantly higher than those treated with PBS. Because TNF-α stimulates cells (MAECs or HFLS-RA cells; Croft et al., 1999) to express VCAM-1, we can conclude from the results that UVHP can target cells with VCAM-1 overexpression.

We further evaluated the targeting capability of UVHP to VCAM-1 with ICP-OES. The above MRI and statistical results have shown that UVHP can indeed target cells that are highly expressing VCAM-1. Cells (MAECs or HFLS-RA cells) were treated with TNF-α for 24 h and then incubated with different concentrations of UVHP (Fe concentration 25, 50, 100, 200 µg/mL). We found that the iron content in both cells was limitedly increased with the increase in UVHP concentration (Figure 4D and Figure 4H). At the same time, compared with the TNF-α treatment groups, the uptake of Fe was also significantly increased under PBS treatment with increasing UVHP concentration, but overall significantly lower than in the TNF-α treatment group with the same UVHP concentration (Figure 4D and Figure 4H). These results further confirmed that UVHP had a targeted function at the cellular level.

### 3.5 RA Model Mice Construction and UVHP Targeting Joint MRI

RA model mice construction details are listed in *Materials and Methods*. The knee joint sections of the model mice were taken for H&E staining and safranin O/fast green staining.

**FIGURE 5:**
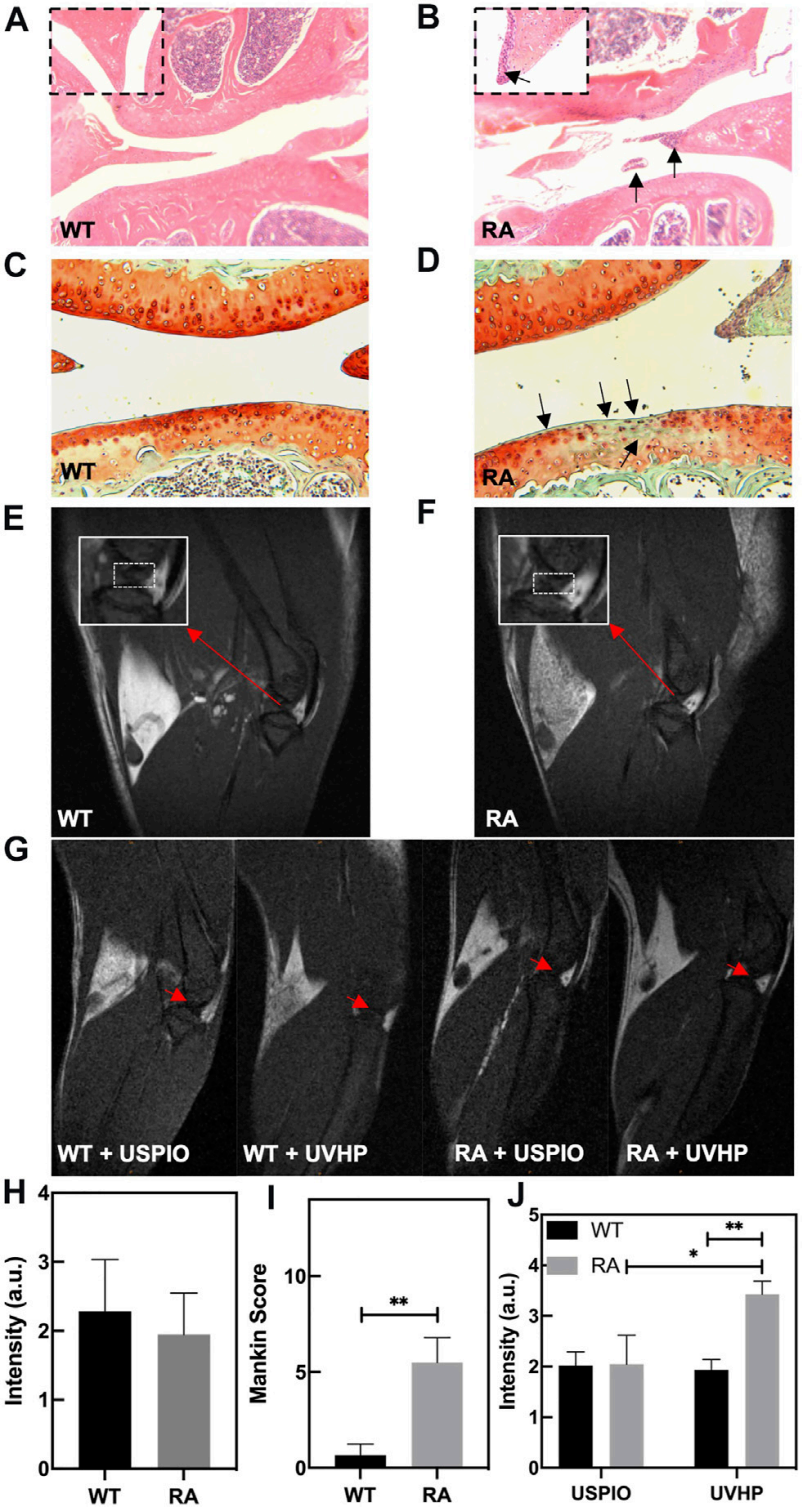
Photomicrographs of representative hematoxylin and eosin (H&E)–stained sections of knee joint from WT mice **(A)** and RA mice **(B)**. The arrow shows the infiltration of inflammatory cells. **(C,D)** Safranin O/fast green staining sections of knee joint from WT mice **(C)** and RA mice **(D)**. The arrow shows the cartilage layer lesion area. **(E,F)** T_1_-weighted MRI of the knee joint in WT and RA mice; red arrows indicate the site of interest. **(G)** T_1_-weighted MRI of the knee joint in WT and RA mice injected with UVHP through the tail vein, at the same time WT and RA mice injected with USPIO in the tail vein as controls; red arrows indicate the site of interest. **(H)** MRI signal value of T_1_-weighted MRI of the knee joint in WT and RA mice. **(I)** Mankin score of knee joints in WT and RA mice. **(J)** Signal value of T1-weighted MRI of the knee joint in WT and RA mice injected with USPIO and UVHP, respectively.

Compared with normal mice (Figure 5A), there are inflammatory cells immersed in the knee joint and the synovium observed in the RA model mice (Figure 5B). The safranin O/fast green staining showed that the cartilage structure of the RA model mice (Figure 5D) was destroyed. The cartilage surface was rough, and the cartilage cells were decolorized. The Mankin score (Figure 5I) of the RA model mice was significantly higher than that of WT mice, and it was in the early stage (Ehrlich et al., 1978). These results indicate that we established the RA model mice successfully.

To confirm the targeting joint MRI effect of UVHP on RA mice, first, we performed T_1_-weighted MRI on normal WT and RA mice, respectively. As shown in Figures 5E, F, the area framed by the dashed line is the synovial fluid region between the joints. Our statistics for this part of the area showed that there was no significant difference between normal WT and RA mice (Figure 5H).

**FIGURE 6:**
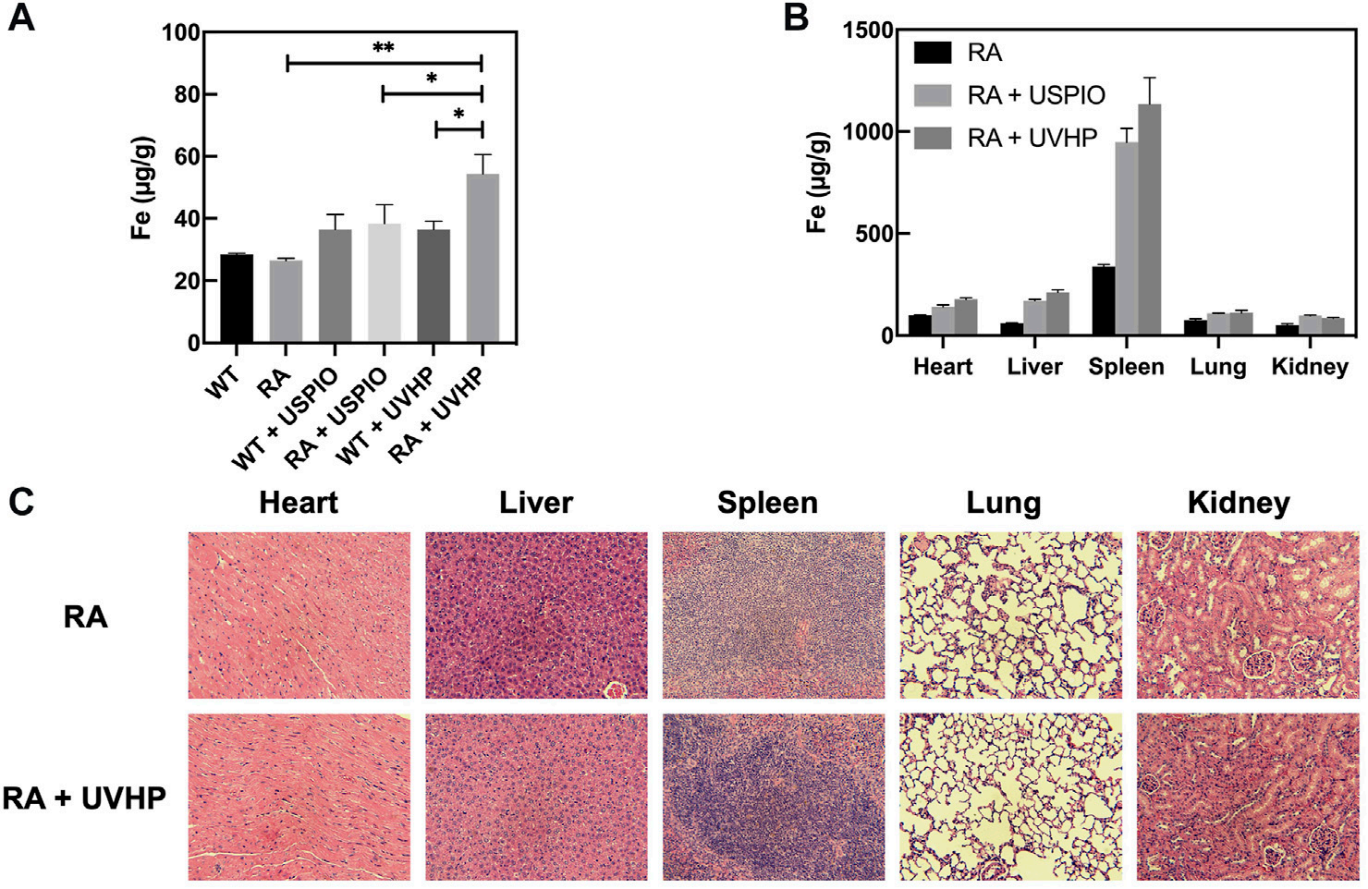
Fe content in different tissues. **(A)** Elemental Fe content in the knee joint 24 h after tail vein injection of USPIO, UVHP to RA mice, WT mice, and PBS-injected RA mice, with WT mice as control. Each group (*n* = 3 per group) was injected with an equal Fe concentration (250 µg Fe, in 0.1 mL PBS). **(B)** Biodistribution of Fe in the major organs (heart, liver, spleen, lung, kidney) in RA mice (n = 3) at 24 h after intravenous administration of the USPIO, UVHP (250 µg Fe, in 0.1 mL PBS). The Fe content of each organ was measured by ICP-OES. RA mice (*n* = 3) injected with PBS served as controls. **(C)** Sections of each organ (heart, liver, spleen, lung, kidney). The tail vein of RA mice (n = 3) was injected with UVHP (Fe contains 250 µg in 0.1 mL PBS), and H&E staining of each organ (heart, liver, spleen, lung, kidney) was performed after 14 days. RA mice (*n* = 3) injected with saline served as control.

Next, we injected USPIO and UVHP to normal WT mice and RA mice via the tail vein, respectively; 24 h later, we performed a T_1_-weighted MRI of the joint area (Figure 5G). The signal values of the synovial region between the joints were counted, and the results are shown in Figure 5J. There was no significant difference in signal values between WT and RA mice after USPIO injection, but the signal values of RA mice were significantly higher than those of WT mice after UVHP injection (Figure 5J). This indicates that more UVHPs were enriched at the joint region of RA mice, whereas USPIO was not enriched at that region. These results proved that UVHP could be targeted to joints of RA mice. To further confirm that UVHP could target the RA region, the changes of Fe content in the knee joint were tested by ICP-OES. The results show that there was no significant difference in Fe content in the knee joints of the WT and RA mice injected with USPIO, but the RA mice injected with UVHP showed a significant increase in Fe content in the knee joints of RA mice (Figure 6A), which was consistent with our MRI results (Figure 5J). This indicates that UVHP could target the joints of the RA model mice.

### 3.6 Biodistribution of UVHP *in vivo*

After injection of USPIO and UVHP via the tail vein to the RA mice 24 h later, these mice were euthanized. The Fe content of the heart, liver, spleen, lung, and kidney was quantified by ICP-OES. The results show that comparing with the order of the Fe distribution in RA mice: the spleen, heart, lung, liver, and kidney (from high to low) (Figure 6B), after injection of USPIO or UVHP to RA mice, the order of the Fe distribution in RA mice was spleen, liver, heart, lung, and kidney. The Fe contents in the spleen and liver of RA mice injected with USPOI or UVHP were significantly higher than those injected with PBS, indicating that most of the USPIO and UVHP were absorbed in the spleen and liver. Reports show more phagocytosis of nanoparticles by macrophages in the liver and spleen (Maldiney et al., 2014), suggesting that RA mice may mainly metabolize UVHP through the liver and spleen.

### 3.7 Safety Evaluation of UVHP

Section pictures of organs stained by H&E were used to investigate the biosafety of UVHP. The morphological analysis (Figure 6C) showed that compared with RA mice injected with PBS, the morphology of major organs (the heart, lung, liver, spleen, and kidney) still showed no significant change after injecting UVHP to RA mice for 14 days, which indicates UVHP had no significant influence on RA mice.

## 4 Discussion

Magnetic iron oxide nanoparticles (>50 nm) are commonly used as T_2_ contrast agents; but when their size is reduced to small diameters (usually <5 nm), the particle surface area increases dramatically, causing relaxation changes of iron nearby water protons (Zhou et al., 2015). Although a large number of studies have explored the potential of USPIO as a T_1_ contrast agent, Shen et al. synthesized a series of USPIO (sizes from 1.9 to 4.9 nm) to explore the optimal size for making T_1_-weighted MRI contrast agents, and the results show that 3.6 nm was the optimal size (Shen et al., 2017). Multiple factors affect T_1_-weighted MRI of USPIO, including size, shape, composition, surface coating, hydrophobicity, and degree of aggregation of USPIO (Jeon et al., 2021). The average size of our synthesized USPIO was 4.7 nm, and the UVHP potential was −27.7 mV with good dispersion, and our results confirm that UVHP targeted not only the cells with high VCAM-1 expression but also the RA mice joint sites, which indicates the potential of UVHP as a T_1_ contrast agent. In the process of USPIO and UVHP being engulfed by cells or entering living tissues, USPIO and UVHP aggregation may occur and lead to enhancement of T_2_-weighted MRI (Roch et al., 2005); thus, we speculate that our synthesized USPIO and UVHP may be suitable for both T_1_- and T_2_-weighted MRI.

Because of the central role of macrophages in the pathogenesis of RA (Mulherin et al., 1996; Kinne et al., 2007), current contrast agents targeting RA imaging are focused on macrophages. Folic acid (Dai et al., 2014) or other molecules were utilized to target inflammatory macrophages in RA area (Hou et al., 2020). Based on the mechanism of inflammatory factors in the synovial fluid of RA patients leading to high VCAM-1 expression in synovial tissue (Carter and Wicks, 2001), we used UVHP targeting VCAM-1 of MAECs in RA. ICP-OES test results proved that UVHPs were enriched in arthritis and may provide a new targeting RA contrast agent for medical MRI.

Most T_1_-weighted contrast agents targeting RA are based on Gd reagents. For example, Hou. et al. synthesized the RA contrast agent CB86-DTPA-Gd targeting translocator protein based on Ga chelates (Hou et al., 2020). The risk of Gd long-term toxicity may trigger nephrogenic systemic fibrosis, as well as Gd deposition in the brain (Todd et al., 2007; Wei et al., 2017). Many studies have demonstrated the biosafety of USPIO, including at the cellular level, such as in hepatocytes (He et al., 2018) and stem cells (Ledda et al., 2020), and at the biological level, such as in mice (Ledda et al., 2020) and rats (Garcia-Fernandez et al., 2020). Through CCK-8 assays, hemolysis assays, and organ H&E staining section assays, we demonstrated that UVHP has good biosafety at the cell and tissue levels.

*In vitro* experiments with cultured human monocytes have shown that various iron oxides have different abilities to be phagocytosed, with SPIO being more readily taken up by human monocytes than USPIO (Metz et al., 2004). *In vivo*, the larger particles (SPIO) are mainly taken up by macrophage cells in the liver, whereas the smaller particles (USPIO) are mainly taken up by macrophages in the bone marrow, lymph nodes, and spleen (Weissleder et al., 1990). Our results show that ultimately only a smaller amount of UVHP is concentrated in the RA lesion area. Analysis on the distribution of UVHP in the organism should also note that UVHP was less concentrated in the RA lesion region in contrast to the liver, spleen, heart, lung, and kidney. Based on the above results, we speculate that USPIO and UVHP may have the function of T_1_-weighted MRI similar to Gd. We look forward to further in-depth research later.

## 5 Conclusion

In summary, we synthesized a simple, low-cost, and less toxic contrast agent UVHP, which targets VCAM-1 and may be useful for early-stage RA diagnosis and generate high contrast in T_1_-weighted MRI.

## Data Availability

The original contributions presented in the study are included in the article/Supplementary Material, further inquiries can be directed to the corresponding authors.
