# Supplementary material for: VHPKQHR Peptide Modified Ultrasmall Paramagnetic Iron Oxide Nanoparticles Targeting Rheumatoid Arthritis for T1-Weighted Magnetic Resonance Imaging
Source: Front Bioeng Biotechnol. 2022 Feb 28;10:821256. doi: 10.3389/fbioe.2022.821256 (PMC8918785; doi:10.3389/fbioe.2022.821256)
Supplement: Supplementary file 1 [file Table1.DOCX]

Supplementary Material

# Fe content of USPIO and UVHP

Supplementary Table 1. Fe content of USPIO and UVHP.

| Sample | Fe content |
| --- | --- |
| USPIO | 44.63 % |
| UVHP | 13.10 % |

The Fe element percentage of USPIO and UVHP was 44.63%, 13.10% respectively. The amount of UVHP and USPIO in subsequent experiments are calculated based on this content. For example, the Fe content is 100 μg, corresponding to UVHP 763.34 μg and USPIO 224.06 μg.

We weighed USPIO 4.1mg, dissolved it with aqua regia until clear (organs and tissues need to be dried in advance), and diluted to 1L with ddH_2_O. The concentration of the sample solution was 4.1 mg/L. The Fe concentration determined by ICP-OES was 1.84 mg/L, so the Fe content (%) of USPIO was 44.63%.
